# Rituximab therapy improves recalcitrant Pemphigus vulgaris

**DOI:** 10.3205/2014-603

**Published:** 2015-01-21

**Authors:** Pedram Noormohammadpour, Amirhooshang Ehsani, Hossein Mortazavi, Maryam Daneshpazhooh, Kamran Balighi, Mohammad Mofidi, Fatemeh Gholamali, Ali Sadeghinia

**Affiliations:** 1 Department of Dermatology, Razi Hospital, Tehran University of Medical Sciences, Tehran, Iran; 2 Bullous Research Center, Tehran University of Medical Sciences, Tehran, Iran

**Keywords:** Pemphigus vulgaris, Rituximab, anti-desmoglein 1 antibody, nti-desmoglein 3 antibody, CD20 positive cells

## Abstract

Pemphigus is a severe life-threatening blistering disease associated with autoantibodies against cell adhesion proteins desmogleins 1 and 3. Patients with severe pemphigus commonly show high rates of relapse after conventional immunosuppressive therapy. The newly developed drug Rituximab showed impressing promises in the treatment of refractory pemphigus vulgaris (PV). In the present study the efficacy of a single course rituximab therapy in the treatment of PV was investigated. Eighteen patients with severe recalcitrant PV were recruited to this study. Pemphigus disease activity index (PDAI), anti-desmoglein 1 and anti-desmoglein 3 antibody titers, and percent of CD20 positive cells were measured at baseline, 10 ± 1, and 22 ± 2 weeks after rituximab therapy. Rituximab was given intravenously at dose 375 mg/m^2^ once weekly for 4 weeks. Rituximab therapy caused a dramatic reduction in the PDAI, accompanied by decreases in anti-desmoglein 1 and anti-desmoglein 3 antibody titers over the follow-up course. The B-cell population decreased at the first follow-up, but returned to its baseline levels at the second follow-up. Rituximab therapy decreased the dose of immunosuppressive drugs required to control the disease. It seems that the rituximab may be effective and safe for treatment of refractory PV.

## Introduction

Pemphigus is a severe, potentially life-threatening autoimmune disease associated with mucous and cutaneous blisters caused by autoantibodies against cell adhesion proteins desmogleins 1 (Dsg1) and 3 (Dsg3). There are two major types of the disease. In pemphigus foliaceus, superficially occurring blisters are typically associated with autoantibodies to the Dsg1, whereas in the more severe pemphigus vulgaris (PV), which is characterized by deeper blisters in the suprabasal layer of the epidermis, IgG antibodies to both the Dsg1 and Dsg3 implicate in pathogenesis of the disease. Binding of antibodies to the desmosomal proteins gives rise to impaired cell-cell adhesion between neighboring keratinocytes and subsequent blister formation (Grando, 2012[14]; Scully and Challacombe, 2002[31]; Waschke, 2008[32]). 

Conventionally, the pemphigus has been treated with corticosteroids and immunosuppressants to decrease antibody production by generally suppressing the immune system. Although the use of these agents dramatically improved the prognosis of the disease, but it also raised the morbidity associated with the long-term use of these drugs, including fatal infection and secondary cancers. This has prompted the development of alternative therapies including the biological agents such as intravenous immunoglobulin and rituximab, as well as interventional treatments such as plasmapheresis and immunoadsorption (Ruocco et al., 2013[30]). 

Rituximab is a potent mouse/human chimeric anti-CD20 monoclonal antibody that selectively targets and depletes antibody-producing B lymphocytes, but spares long-lived plasma cells that are resident in the bone marrow and represent the major source of serum antibodies. Consequently, rituximab induces a decline of circulating anti-desmoglein autoantibodies. Additionally, it leads to a decrease in desmoglein-specific auto-reactive T cells. Therefore, rituximab has been reported to be beneficial in treating PV (Bomm et al., 2013[6]; El Tal et al., 2006[11]; Zambruno and Borradori, 2008[33]). 

In order to further explore the efficacy of rituximab therapy in PV patients, we conducted a prospective study in which the effect of rituximab administration on the clinical outcome, Dsg1 and Dsg3 antibody titers, and percent of CD20 positive cells was assessed in a group of PV patients in Iran. Considering the extremely high price of rituximab (Marzano et al. , 2007[22]), making a medical decision on prescribing the therapy requires a satisfactory level of optimism toward its clinical efficacy, assuring a prolonged, safe, and cost-effective outcome. Here we report promising results of our study, which together with those previously reported, may help dermatologists to make a better decision on the approaches for managing severe corticosteroid- and immunosuppressive-refractory PV.

## Methods

This prospective study was conducted at the Razi Hospital, Tehran, Iran from 2012 to 2013. The study protocol was approved by the ethics committee of Tehran University of Medical Sciences. Patients with severe refractory PV were enrolled in the study. The diagnosis of PV was made on the basis of typical clinical presentation and laboratory evaluations, including histological and immunological criteria (Harman et al., 2003[17]). The refractoriness of the disease was defined as recurrent blister formation despite immunosuppressive therapy with prednisolone >10 mg daily and/or immunosuppressants (Azathioprine 2 mg/kg daily or equivalent immunosuppression) (Marzano et al., 2007[22]). Written informed consents were obtained from all participants. All study patients had a clear indication for rituximab treatment, and none of them had a severe cardiac disease, hepatic or renal disorder, active infection, immunodeficiency, or cancer. 

Basic demographic information on the patients’ age and gender was collected. The baseline clinical status of pemphigus, defined as pemphigus disease activity index (PDAI) (Grover, 2011[15]; Murrell et al., 2008[25]) previously proved to be the most valid scoring system in the assessment of pemphigus (Rahbar et al., 2014[28]), and the dosage of the prescribed corticosteroid and immunosuppressants were recorded. Anti-Dsg1 and anti-Dsg3 autoantibodies were titered by enzyme-linked immunosorbent assay [EUROIMMUN, Germany]. Percent of CD20 positive blood cells was measured using flow cytometry [kit: Daco, Denmark; device: Partec PAS, Germany]. Then, all patients were treated with intravenous rituximab 375 mg/ m2 body surface once weekly for 4 consecutive weeks. The severity of pemphigus, the dosage of corticosteroid and/or immunosuppressants, the anti-Dsg1 and anti-Dsg3 antibody titers, and the percent of CD20 expressing cells were evaluated, on average, 10 (± SEM: 1) and 22 (± 2) weeks following the rituximab therapy. The dosage of corticosteroid was tapered by approximately 30 % when a minimum reduction of 30 % was observed in the PDAI score relative to the previous evaluation. Only patients who were accessed for both of the follow-up assessments were ultimately included in the data analysis. 

Data were analyzed using IBM SPSS statistics 20. The normal distribution of data was assessed using the Shapiro-Wilk W test. Since the distributions of anti-Dsg1 and anti-Dsg3 antibody titers and CD20 positive fraction violated the normal fitting, non-parametric tests were used for subsequent data analysis. Friedman’s test was applied to explore overall significant differences in the PDAI, anti-Dsg1 and anti-Dsg3 antibody titers, and percent of CD20 positive cells over the three evaluation occasions. To examine where the differences actually occurred, separate Wilcoxon signed-rank tests were applied to the different combinations of related groups. The Spearman rho test was utilized to explore associations between the PDAI and anti-Dsg1, anti-Dsg3, and CD20 positive fraction. Data are presented as median (interquartile range). Two-tailed p values less than 0.05 were considered significant.

## Results

A total of 24 patients with recalcitrant PV were enrolled in the study and treated with rituximab, from which 18 patients (13 men and 5 women) attended both of the follow-up sessions and included in the final analysis. Among these patients, 5 had mucosal and 13 had mucocutaneous PV. The median (interquartile range) age of the patients was 33.5 (26‒46.5) years. At the outset of the study, all of the patients were on prednisolone therapy 20‒90 mg daily, and 9 of them were concurrently receiving immunosuppressants (2 patients: mycophenolate mofetil 2 g daily; 4 patients: azathioprine 100 mg daily; and 3 patients: methotrexate 7.5‒10 mg weekly). The baseline and post-rituximab PDAI score, anti-Dsg1 and anti-Dsg3 antibody titers, and CD20 positive cells fraction are presented in Table 1(Tab. 1). 

The PDAI showed a significant decrease over the rituximab treatment and follow-up course [Friedman's test: *Χ^2^* (2) = 32.44, *p* < 0.001]. Wilcoxon signed ranks tests revealed that the differences occurred at both the first (*Z* = -3.73, *p* < 0.001) and the second follow-up evaluations (*Z* = -3.72, *p* < 0.001 compared with the baseline; *Z* = -2.37, *p* = 0.02 compared with the first follow-up) subsequent to rituximab therapy. All cases showed a dramatic decrease in their PDAI at the first follow-up, and except for 2 cases, all of the patients had a lower PDAI at the second follow-up relative to the first one.

**Figure 1:**
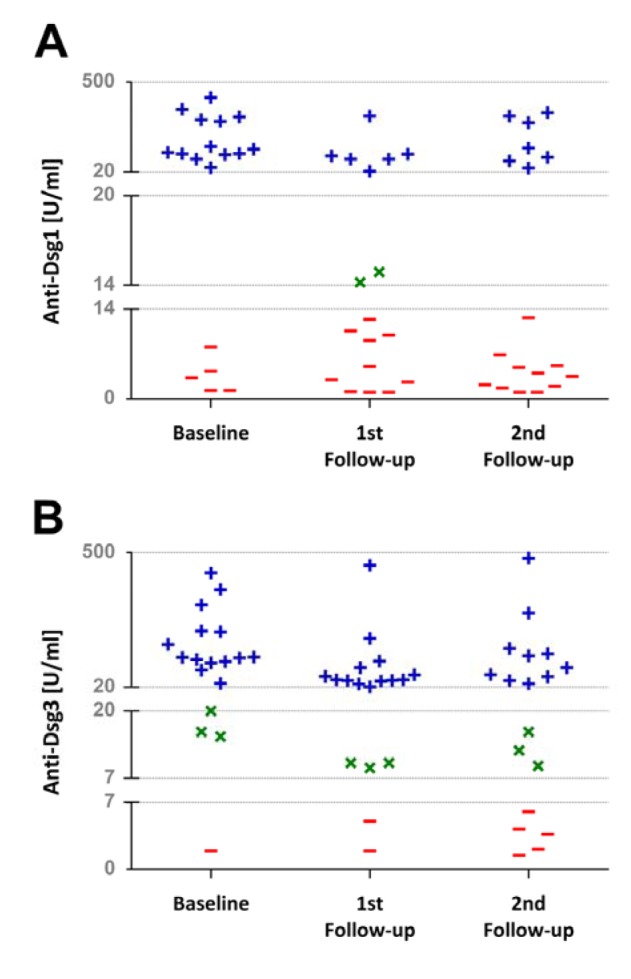
Scatterplot showing the number of patients with negative (-), intermediate (×), and positive (+) anti-Dsg1 (A) and anti-Dsg3 (B) antibody titers over the rituximab treatment and follow-up course. Apparently, rituximab therapy decreased the number of patients with positive anti-Dsg1 and anti-Dsg3 titers. The gridlines represent the lower and upper limits of negative, intermediate, and positive titers.

There was a significant overall change in anti-Dsg1 antibody titer over the treatment and follow-up course [Friedman's test: *Χ^2^* (2) = 12.19, *p* = 0.002]. Pairwise comparisons revealed that the antibody titer decreased significantly 10 weeks after the rituximab treatment relative to the baseline levels (*Z* = -3.33, *p* = 0.001), but there was no significant change occurred at the second follow-up occasion (*Z* = -1.76, *p* = 0.07 compared with the baseline; *Z* = -0.16, *p* = 0.86 compared with the first follow-up). Moreover, an overall significant difference was found in the anti-Dsg3 antibody titer over the treatment and follow-up period [Friedman's test: *Χ^2^* (2) = 11.74, *p* = 0.003]. Post hoc analysis with Wilcoxon test showed that the anti-Dsg3 antibody decreased after the rituximab treatment (*Z* = -3.10, *p* = 0.002) but remained stable over the later follow-up course (*Z* = -2.41, *p* = 0.01 compared with the baseline; *Z* = -0.41, *p* = 0.68 compared with the first follow-up). Rituximab therapy decreased the number of patients with positive levels of anti-Dsg1 and anti-Dsg3 antibodies (Figure 1(Fig. 1)).

The proportion of CD20 positive cells exhibited a significant decrease over the treatment and follow-up period [Friedman's test: *Χ^2^* (2) = 12.02, *p* = 0.002]. Further analysis showed that the percent of CD20 positive cells decreased after rituximab therapy (*Z* = -2.37, *p* = 0.02) but started to increase again at the second follow-up (*Z* = -1.89, *p* = 0.06 compared with the baseline; *Z* = 2.00, *p*= 0.04 compared with the first follow-up). 

Analysis of the data with Spearman rho test showed that the PDAI was significantly correlated with anti-Dsg1 and anti-Dsg3 titers on almost all measurements, but not with the CD20 positive cell fraction (Table 2(Tab. 2)). 

The median doses of prednisolone before rituximab administration and at the two follow-up occasions were as follows, respectively: 40 (35‒62.5), 30 (20‒32.5), and 18.75 (16.87‒25.62) mg/day. Statistical analysis showed that the required dose of corticosteroid therapy to control the adverse effects of pemphigus declined significantly following the rituximab therapy [Friedman's test: *Χ^2^* (2) = 32.11, *p* < 0.001]. Pairwise comparisons revealed that the need for corticosteroid administration was decreased at the both follow-up sessions (first follow-up: *Z* = -3.63, *p*< 0.001; second follow-up: *Z* = -3.73, *p*<0.001 compared with the baseline; *Z* = -2.99, *p* = 0.003 compared with the first follow-up). However, at the end of the study, the dose of immunosuppressants was decreased in 4, remained fixed in 3, and increased in 2 patients. Additionally, one patient started to receive mycophenolate mofetil. 

## Discussion

The main finding of this study was that rituximab treatment improved severe refractory PV, as demonstrated with decreases in the PDAI, anti-Dsg1 and anti-Dsg3 antibody titers, percent of CD20 positive cells, and required corticosteroid dose to control the disease. 

Several lines of evidence indicated that rituximab therapy may be promising for improving severe refractory PV. In this regard, Arin et al. reported that a single course rituximab treatment elicited clinical improvement in 5 patients with refractory pemphigus over a follow-up period of up to 3 years, allowing immunosuppressive treatment to be reduced or terminated (Arin et al., 2005[2]). Moreover, Pfütze et al.[27] reported that rituximab therapy in 5 patients with mucosal PV induced excellent clinical responses which were associated with a significant reduction in prednisolone dosage and a decrease in anti-Dsg-specific IgG. The patients had no or only minimal residual symptoms over a 12-month period after the therapy (Pfütze et al., 2009[27]). Joly et al.[19] reported that from 21 patients with recalcitrant PV, who were treated with 4 weekly infusions of 375 mg of rituximab per square meter of body-surface area, 19 patients displayed complete remission of the disease at 3 months (Joly et al., 2007[19]). Furthermore, Cianchini et al. reported that treatment with the same regimen of rituximab in patients with refractory PV and pemphigus foliaceus resulted in a good clinical response during an 18-month follow-up period, along with a consensual decline of the serum antidesmoglein titers (Cianchini et al., 2007[8]). Additionally, it has been shown that patients treated with rituximab earlier in the disease course may have better outcomes, enduring complete remission with no or minimal systemic therapy for 19 months (Lunardon et al., 2012[21]). Other studies also indicated that adjuvant rituximab therapy may hold promises in long-lasting treatment of severe recalcitrant PV with no or slight adverse drug-related events (Baum et al., 2013[4]; Behzad et al., 2012[5]; Cho et al., 2014[7]; Corral et al., 2013[9]; Craythorne et al., 2011[10]; El Tal et al., 2006[11]; Eming et al., 2008[12]).

In line with the previous studies, we have shown that a single cycle rituximab therapy together with conventional immunosuppressive treatment resulted in remarkable clinical improvements in patients with severe corticosteroid- and immunosuppressant-recalcitrant PV. The clinical improvement was accompanied by a decrease in anti-Dsg1 and anti-Dsg3 titers, as demonstrated by a significant association between the PDAI and antibodies against the Dsg1 and Dsg3, which suggests that these autoantibodies implicate in pathogenesis of the pemphigus. However, anti-Dsg1 and anti-Dsg3 titers remained positive in 36 and 64 percent of the patients, respectively. This is in agreement with other studies showing decreases in the anti-Dsg1 and anti-Dsg3 antibodies subsequent to rituximab therapy (Muller et al., 2010[24]; Pfütze et al., 2009[27]; Reguiai et al., 2012[29]), but still positive titers in most of the patients (Reguiai et al., 2012). The persistence of circulating anti-Dsg1 and anti-Dsg3 autoantibodies during the remission of PV is still not fully understood. It might be explained by the expression of nonpathogenic epitopes on the Dsg1 and Dsg3 ectodomains, causing defective interaction with the fatty acid signaling complex ligand strongly implicated in acantholysis development (Amagai et al., 2006[1]).

What is more, after an initial remarkable decline, anti-Dsg1 and anti-Dsg3 antibodies followed seemingly different time courses, as the former began to return to its baseline value approximately 22 weeks after rituximab therapy, while the latter continued to dwindle over time. Indeed, anti-Dsg1 also exhibited a decrease at the second follow-up, but it did not reach significance only with a trivial shortcoming (*p* = 0.07), mainly because of some increases occurred in 6 out of 18 patients. Nevertheless, parts of B-cell population might have been preferentially affected by rituximab. It has been shown that at least five to six different B-cell clones may be involved in producing pathogenic anti-Dsg3 antibodies and rituximab may have different effects on these clones. As a result, some of these clones may shutdown later than others and continue to produce some anti-Dsg3 antibodies for a while (Hammers et al., 2014[16]). This may describe different response of anti-Dsg1 and anti-Dsg3 antibodies to treatment with this drug. Moreover, rituximab was found to selectively reduce anti-Dsg1 and anti-Dsg3 antibodies while causing significant increases in anti-VZV and anti-EBV antibodies through different effects on B-cell activating factor (BAF) and a proliferation inducing ligand (APRIL) (Nagel et al., 2009[26]). This may further elucidate possible diverse effects of rituximab on different cell population and even on different B-cell clones.

Percent of CD20 positive cells was not connected with the disease activity, even though the CD20 fraction showed significant reductions on the first follow-up after the rituximab therapy relative to the baseline levels. This may imply that the rituximab effect is not mediated only via B-cell depletion (Zambruno and Borradori, 2008[33]). What is more, CD20 fraction returned to the baseline levels at the second follow-up (22 weeks after rituximab therapy), indicating repopulation of B-lymphocytes in the presence of clinical remission. However, Mouquet et al. showed that the B-cell population depleted days after rituximab therapy, remained in complete depletion for 6 months, started to reconstitute between month 6 and 9, and never reached to the baseline levels until 2 years (Mouquet et al., 2008[23]). The reason for this discrepancy is not clear to us. However, it might be due to the different way of monitoring B-lymphocytes, in that, they measured CD19^+^ B-cells, whereas in our study we measured CD20^+^ B-cells. It should be noted that assessment of CD20 by flow cytometry may be impaired in the presence of rituximab in the plasma (Arin et al., 2005[2]).

Regarding the dosage of rituximab therapy, we used a regimen previously applied in other studies, namely 375 mg/m^2^ body surface once weekly for four consecutive weeks (El Tal et al., 2006[11]; Feldman et al., 2012[13]; Marzano et al., 2007[22]; Muller et al., 2010[24]; Pfütze et al., 2009[27]; Reguiai et al., 2012[29]). This regimen appears to be effective in approximately all of these studies. In our study, all of the patients treated with this dose showed excellent clinical improvements. Only 2 patients showed partial increases in their disease activity at the second follow-up compared to the first evaluation. Consistent with this, it has been shown that pemphigus patients treated with 3 or more infusions of rituximab at dose 375 mg/m^2^ demonstrated better outcomes in time to complete remission and relapse rate relative to those treated with 2 infusions of rituximab at the same dose (Kim et al., 2011[20]). Additionally, in a previous study performed in our department, the same regimen was used for treating 40 PV patients, all of which showed an initial clinical improvement 6.35 weeks following the rituximab treatment and a marked clinical improvement after a mean of 10.13 months (Balighi et al., 2013[3]). However, a fixed-dose rheumatoid arthritis regimen of rituximab (1 g intravenously on days 1 and 15, followed by 500 mg intravenously if clinically warranted at 6-month intervals or repeated full dosing) was recently proved to be efficacious and well tolerated in patients with PV, and proposed for administration in patients who do not achieve remission after one cycle or patients who experience relapse (Heelan et al., 2014[18]). Side effects of rituximab include systemic infections, deep venous thrombosis, long-term reductions in the plasma levels of gammaglobulin, and neutropenia (Ruocco et al., 2013[30]). None of the patients in our study exhibited these adverse effects, which implies the safety of the treatment protocol. 

One limitation of this study was the lack of a control group of patients with no rituximab therapy due to ethical considerations. However, it should be reminded that the patients recruited to this study were non-responder to conventional immunosuppressive-based treatment regimens, which obviated the need for including control subjects. An imaginable solution to this drawback could be assessing retrospectively the data from patients with recalcitrant PV disease but with no alternative treatment. Indeed, one study followed up clinically and immunologically severe PV patients treated with or without rituximab (Reguiai et al., 2012[29]). However, it failed to find out any significant superiority for rituximab therapy over the immunosuppressive alone therapy in terms of long-term remission rate and immunologic profile of anti-Dsg1 and anti-Dsg3 titers. 

In conclusion, this study highlights the prolonged effect and disease control after one single course of rituximab therapy. The clinical improvement was associated with decreases in the doses of corticosteroids and immunosuppressants, and accompanied with reductions in anti-Dsg1 and anti-Dsg3 antibodies. Percent of CD20 positive cells declined at the first follow-up, but recovered later at the second follow-up. No adverse effect related to the rituximab was observed among the study patients. Taken together, it seems that rituximab may be effective and safe for treatment of refractory PV.

## Acknowledgements

This work was supported by a grant from Tehran University of Medical Sciences.

## Declaration of interest

There is no conflict of interest for this work. 

## Figures and Tables

**Table 1:**
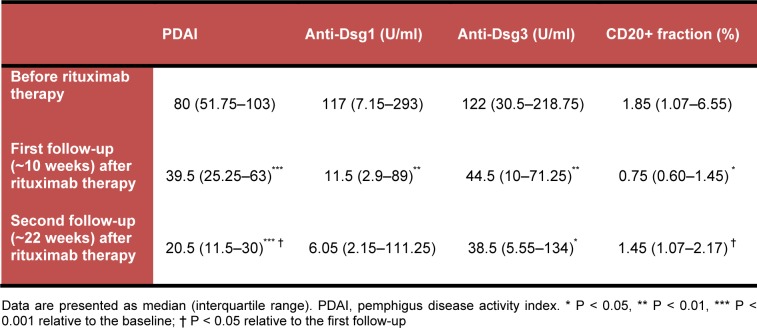
Baseline and post-rituximab status of PDAI, anti-Dsg1 and anti-Dsg3 antibody titers, and CD20 positive cells fraction

**Table 2:**
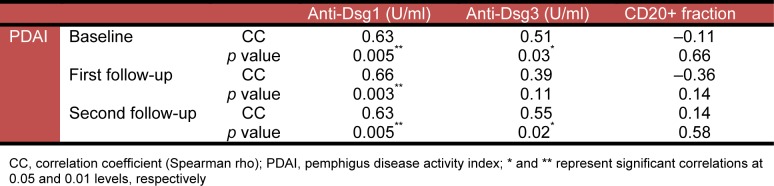
Correlation between the PDAI and anti-Dsg1, anti-Dsg3, and CD20 positive cells
